# Biology, General and Medical

**Published:** 1911-01

**Authors:** 


**Biology, General and Medical.** By **Joseph McFarland,** M.D., Professor of Pathology and Bacteriology in the Medico-Chirurgical College of Philadelphia. 12 mo, pp. 440. With 160 illustrations. Philadelphia and London: W. B. Saunders Co. 1910. (Cloth, $1.75.)

McFarland is well known to the medical profession as a teacher and writer and has written a work on biology that compares very well with his other books on kindred subjects. The volume consists of eighteen chapters, all of which are of interest to the medical student, as all problems of biological science are in a sense medical. Chapter XV, which deals with infection and immunity is most interesting and practical and leaves but little unsaid on that subject. The same may be said of chapter XVII, which takes up the subject of grafting. The illustrations and general makeup of the book are in keeping with the modern art of bookmaking. J. A. R.

